# Molecular Identification of *Anopheles* (Diptera: Culicidae) Species in Native Communities of a Northeastern Region of Peru

**DOI:** 10.3390/microorganisms13040861

**Published:** 2025-04-10

**Authors:** Eddyson Montalvo-Sabino, Marianella Villegas-Pingo, Jhon Zumaeta, Lizandro Gonzales, Rafael Tapia-Limonchi, Marta Moreno, Christian R. González, Stella M. Chenet

**Affiliations:** 1 Instituto de Investigación de Enfermedades Tropicales, Universidad Nacional Toribio Rodríguez de Mendoza de Amazonas (UNTRM), Chachapoyas 01001, Peru; eddysonmont@gmail.com (E.M.-S.); keyko.villegas@untrm.edu.pe (M.V.-P.); jhon.zumaeta@upch.pe (J.Z.); rafael.tapia@untrm.edu.pe (R.T.-L.); 2 Laboratorio Referencial Regional de Salud Pública Amazonas, Dirección Regional de Salud (DIRESA), Chachapoyas 01001, Peru; gonzalesc@hotmail.com; 3 Facultad de Medicina (FAMED), Universidad Nacional Toribio Rodríguez de Mendoza de Amazonas (UNTRM), Chachapoyas 01001, Peru; 4 Department of Infection Biology, London School of Hygiene & Tropical Medicine, London WC1E 7HT, UK; marta.moreno@lshtm.ac.uk; 5 Instituto de Entomología, Universidad Metropolitana de Ciencias de la Educación, Santiago 7760197, Chile; christian.gonzalez@umce.cl

**Keywords:** *Anopheles*, *Plasmodium*, malaria, *Cox1* barcode, Amazonas

## Abstract

Background: Malaria is a severe health problem in native communities of Condorcanqui in the Amazonas region of Peru. Recently, the number of malaria cases has increased considerably following a *Plasmodium falciparum* outbreak in 2019. However, there is no information on the anophelines acting as *Plasmodium* vectors in this area. This study aimed to identify *Anopheles* species circulating in previously unexplored native communities of Condorcanqui. Additionally, we sought to detect the presence of DNA from *P. vivax* and *P. falciparum* parasites in mosquitoes. Methods: During three exploratory visits between March and September 2022, 453 mosquitoes were collected using Shannon traps and CDC light traps. Only specimens morphologically identified as *Anopheles* sp. were subjected to molecular confirmation through PCR amplification and sequencing of the *Cox1* barcode region. *Plasmodium* parasites were detected using nested PCR targeting of the 18S rRNA subunit, while human blood meal feeding was evaluated using a human *β-globin* marker. Results: A total of 66 specimens were molecularly confirmed as anopheline species: *An. benarrochi* B, *An*. *triannulatus*, *An*. *Costai*, and *An. nimbus*. Six specimens of *An. benarrochi* B were exclusively positive for *Plasmodium* parasites by PCR. Moreover, four specimens tested positive for *Plasmodium* and the presence of human blood, suggesting the anthropophilic behavior of *An. benarrochi* B and its possible role as a potential malaria vector in this area. Conclusions: In conclusion, while this study provides valuable insights into the potential role of *Anopheles benarrochi* as a malaria vector in Amazonas, further research is essential to fully understand its behavior and transmission dynamics in the region.

## 1. Background

Malaria is a potentially fatal disease caused by *Plasmodium* parasites, transmitted through the bites of infected *Anopheles* mosquitoes. In 2022, it was estimated that there were 249 million cases and around 608,000 deaths worldwide [1]. Peru reported 26,217 malaria cases in 2024, caused by *Plasmodium vivax* (82%) and *P. falciparum* (18%) infections [2]. Loreto is the most affected region in the country, accounting for 94% of cases [2]. Amazonas, a northeastern region of Peru, is the third most affected area and has reported a notable increase in the number of malaria cases in recent years. Approximately 90.6% of the cases are reported in the district of Río Santiago, Condorcanqui [3], a remote and extremely impoverished area where most native communities reside along the riverbanks with restricted medical care and no access to electricity or potable water [4].

In Peru, at least nine species of *Anopheles* have been identified as malaria vectors. Among these, four are considered primary vectors: *Anopheles (Nyssorhynchus) darlingi* Root, *An. (Nys) benarrochi* Gabaldón, Cova García, and López, *An. (Nys) albimanus* Wiedemann, and *An. (Anopheles) pseudopunctipennis* Theobald; the other five species are classified as secondary vectors [5,6,7,8,9]. Additionally, it has been reported that *An. darlingi* and *An. benarrochi* B are primarily distributed throughout the Peruvian Amazon [7,9].

Identifying the vectors involved in *Plasmodium* transmission is essential for implementing control campaigns. However, the complexity of morphological identification, combined with the high biodiversity and intraspecific variability of mosquitoes, hinders accurate identification [10,11,12,13], leading to significant errors in species classification [14]. Currently, DNA sequencing is crucial for species identification and genetic variation analysis [14,15]. The mitochondrial *Cytochrome c oxidase subunit 1* (*Cox1*) gene is particularly useful for phylogenetic reconstruction and the study of geographic variability within species complexes [12]. This study aimed to identify *Anopheles* species present in native communities of Río Santiago and assess their potential role as malaria vectors through the molecular detection of *Plasmodium* DNA and human blood meals.

## 2. Methods

### 2.1. Study Site

The Amazonas region, located in northeastern Peru, has seven provinces, with Condorcanqui being the one located in the northern sector. This province borders Ecuador to the north and Loreto to the east. Condorcanqui has an area of 17,984.29 km^2^ and is organized into three districts: Nieva, Río Santiago, and Cenepa. It has a humid tropical climate, with temperatures that can reach up to 35 °C and a relative humidity greater than 90%. Furthermore, 95% of its inhabitants belong to the Awajún and Wampis ethnic groups [4].

**Figure 1:**
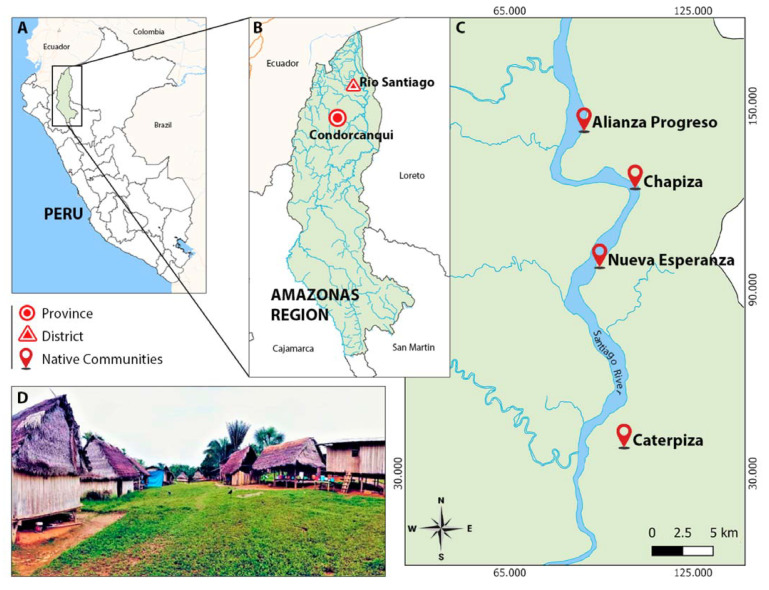
(**A**) Map of Peru and geographical location of the Amazonas region. (**B**) Map of Amazonas indicating the province of Condorcanqui and the district of Río Santiago. (**C**) Mosquito collection sites in the native communities of the Río Santiago district. (**D**) Photo of the native community of Alianza Progreso, 2022.

Mosquitoes were collected in four native communities in the Río Santiago district: Alianza Progreso (AP), Nueva Esperanza (NE), Chapiza (CH), and Caterpiza (CT) (Figure 1) during three visits between March and September 2022. These communities were selected due to a notable increase in malaria cases in recent years. The Chapiza Health Facility, which serves these communities, reported an Annual Parasite Index (API) of 122.8 in 2020, which rose to 224.8 in 2022, with infections caused by both *P. falciparum* and *P. vivax*.

### 2.2. Mosquito Collection

Adult mosquitoes were captured from 18:00 to 06:00 using two CDC light traps and three Shannon traps placed at equidistant points approximately 10 m from the houses. *Anopheles* mosquitoes were morphologically identified to the genus level in the field using entomological keys [16] and stored in 1.5 mL cryovials containing 70% ethanol for subsequent molecular processing.

### 2.3. Molecular Methods for Anopheles Species Identification

Genomic DNA was extracted from whole mosquito bodies using the DNeasy Blood & Tissue Kit (Qiagen, Hilden, Germany), following the manufacturer’s instructions. The 710 bp barcode region of the *Cox1* gene was then amplified using LCO1490 and HCO2198, as described by Folmer et al. [17]. Each 25 μL PCR reaction contained 1 μL of extracted DNA, 0.5 μM of each primer, 1 unit of Platinum Taq DNA polymerase (Invitrogen, Waltham, MA, USA), 0.2 mM of each deoxynucleotide triphosphate, 1X PCR buffer (Invitrogen, Waltham, MA, USA), 2.5 mM MgCl_2_, and nuclease-free water. The amplification conditions were set according to Linton et al. [18].

Appropriate controls were included in all of the PCR assays. An extraction control was used to monitor potential contamination during DNA isolation. No-template controls (NTCs) were included in each PCR run to detect contamination in reagents. A negative control was incorporated to ensure assay specificity. As a positive control, DNA from a previously sequenced and confirmed culicid mosquito was used to validate the amplification.

PCR products were visualized on 2% agarose gels stained with SafeView™ Classic (Applied Biological Materials, Richmond, BC, Canada). Amplicons were purified using the Exo-CIP™ kit (New England Biolabs, Ipswich, MA, USA) and sequenced with the BigDye™ Terminator kit (Thermo Fisher Scientific, Waltham, MA, USA). The primers for sequencing were the same as those used in the PCR. The amplified products were sequenced by capillary electrophoresis on the 3500 Genetic Analyzer (Applied Biosystems, Waltham, MA, USA).

### 2.4. Human Blood Meal Identification and Plasmodium Detection in Mosquitoes

The assessment of human blood meal was conducted using a previously reported procedure for human *β-globin* [19]. The amplification conditions consisted of an initial denaturation at 94 °C for 7 min, followed by 35 cycles of denaturation at 94 °C for 1 min, annealing at 53 °C for 1 min, and extension at 72 °C for 1 min. A final extension step at 72 °C for 5 min concluded the PCR reaction.

The nested PCR technique targeting the 18S rRNA subunit, as described by Singh et al. [20], was employed to detect *P. falciparum* and *P. vivax* parasites in mosquitoes. As a positive control for the human *β-globin* assays, a DNA sample extracted from human blood was used. For *Plasmodium* PCR assays, positive controls from *P. falciparum* (3D7 strain) and *P. vivax* (Sal1 strain) were included.

### 2.5. Bioinformatic Analysis

The sequence data were analyzed using Geneious Prime software v. 2022.2.1 and the Basic Local Alignment Search Tool (BLAST), with searches conducted against the GenBank database to identify the species. Anopheline sequences were aligned using the MUSCLE tool in MEGA X software v. 7.2.6.1.

For phylogenetic analysis, 41 previously reported sequences from different anopheline species were incorporated [6,21,22,23,24]. A Maximum Likelihood (ML) tree was constructed using the Kimura 2-parameter model (K-2P) and the Bootstrap method with 1000 replicates in MEGA X software v. 7.2.6.1 [25]. The *Aedes aegypti Cox1* sequence (NC035159) was used as the outgroup.

## 3. Results

A total of 453 mosquitoes were captured, and 66 specimens were confirmed to belong to the *Anopheles* genus, all of them females (Table 1). *Anopheles benarrochi* B was the predominant species (92,4%, 61/66), followed by *An. triannulatus* (Neiva & Pinto) (3%, 2/66), *An*. *costai* da Fonseca & da Silva Ramos (3%, 2/66) and *An. nimbus* (Theobald) (1.6%, 1/66) (Table 1). The sequences obtained were deposited in GenBank (accession numbers OR729604–OR729669).

**Figure 2:**
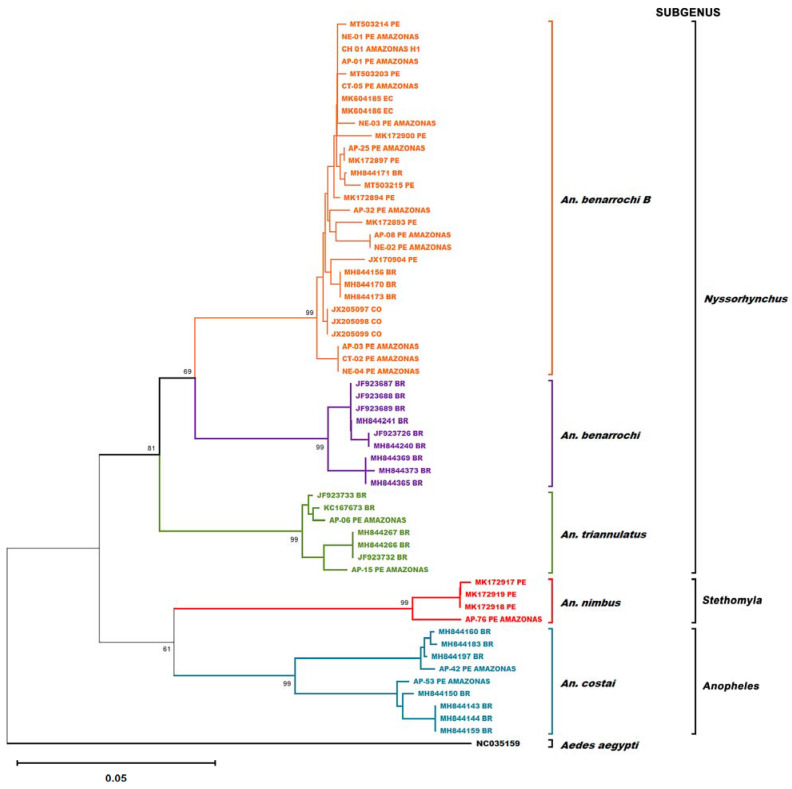
Maximum likelihood (ML) phylogenetic tree using the *Cox1* gene with a total of 58 sequences. Sequences generated in this study are labeled as “PE AMAZONAS”, while reference sequences are denoted as follows: Ecuador (“EC”), Brazil (“BR”), Colombia (“CO”), and other Peruvian sequences not originating from this study as “PE”.

The ML tree based on the K-2P model (Figure 2) corroborated the accurate identification of specimens in this study. *Anopheles benarrochi* B formed a stable monophyletic clade with sequences from Loreto and other countries and a paraphyletic clade with other *An. benarrochi*. The phylogenetic classification aligned with the traditional subgenus groupings: *An. benarrochi* B, *An. benarrochi*, and *An. triannulatus* belong to the *Nyssorhynchus* subgenus; *An. nimbus* to the *Stethomyia* subgenus; and *An. costai* to the *Anopheles* subgenus, as previously documented [26].

Among the 66 anophelines, 23 samples of *An. benarrochi* B and 1 sample of *An. triannulatus* were positive for human *β-globin*, proving that they feed on human blood. Furthermore, the DNA of *Plasmodium* parasites was detected in six specimens of *An. benarrochi* B, with four samples testing positive for *P. falciparum* and two for *P. vivax*.

## 4. Discussion

In the eastern region of Peru, *An. darlingi* is the primary vector of malaria [8]. Additionally, *An. triannulatus* has been reported as the predominant vector in eastern Loreto, while *An. benarrochi* is primarily found in the western part of that region [8,27]. Our study is the first molecular report of anopheline species in native communities of Condorcanqui in the Amazonas region [28]. A total of 66 anophelines were collected, and through sequencing of the barcode region and phylogenetic analysis (Figure 2), 4 species were identified: *An. benarrochi* B, *An. triannulatus, An. costai*, and *An. nimbus. An. benarrochi* B was the most abundant species (*n* = 61), and 23 individuals, along with 1 *An. triannulatus*, showed human blood feeding, indicating anthropophilic behavior, as previously reported [5,9].

*Anopheles benarrochi* s.s. and *An. benarrochi* B are part of a species complex [13,14,24]. *Anopheles benarrochi* B has been strongly implicated as one of the primary vectors of *Plasmodium* in Peru [5,8,9,29], and its vectorial role has been attributed to geographic areas where *An. darlingi* is absent [30]. However, its importance as a malaria vector is not clear, as it is based on circumstantial evidence or the identification of circumsporozoite protein (CSP) of *P. vivax* (genotypes VK210 and VK247) and *P. falciparum* [6,8]. In our study, we identified *Plasmodium* DNA in six specimens of this species, with four samples testing positive for *P. falciparum* and two for *P. vivax*. These mosquitoes were collected in Alianza Progreso, a native community further north than the four previously studied. Although this study has a limited number of samples, the identification of *Plasmodium* DNA in *An. benarrochi* B, its anthropophilic and domestic behavior [6], along with the increase in malaria cases in the studied native communities suggest its role as a vector of *Plasmodium* in the region. Additionally, the detection of human *β-globin* in 23 *An. benarrochi* B specimens reinforces its anthropophilic nature and potential role in malaria transmission. This finding further supports the need for continued entomological and epidemiological studies to assess *An. benarrochi* B vectorial capacity in the region.

The phylogenetic tree showed that the sequences of *An. benarrochi* B were closely related to those from Ecuador, Colombia [24], Peru [24,31], and Brazil [32]. This confirms the identity of the collected specimens and demonstrates the broad geographic distribution of the species, suggesting gene flow between these countries.

Although *An. triannulatus* primarily feeds on animals (zoophilic), it has also been suggested as a vector of *Plasmodium* given its role in Brazil [33,34,35], Peru [36], and Colombia [37]. This species is part of a complex with *An. halophylus* and the putative species *An. triannulatus* C, that may differ in vectorial capacity [12,37].

*Anopheles costai* and *An. nimbus*, which belong to the subgenera *Anopheles* and *Stethomyia*, respectively, are not considered malaria vectors [26,38]. However, *An. costai* feeds on human blood and may be confused with *An. mediopunctatus*, a species implicated as a potential vector of *Plasmodium* [26,39].

Further longitudinal studies should explore seasonal variations in *Anopheles* species composition and malaria transmission dynamics. Emphasis on sporozoite detection would help to confirm vector competence, while blood meal analysis could provide insights into host preferences and transmission patterns in the region.

This study represents the first report of *An. benarrochi* B as a possible malaria vector in the native communities of Condorcanqui. However, the limited number of samples highlights the need for further research to assess the involvement of other *Anopheles* species.

## 5. Conclusions

In conclusion, this study represents the first molecular investigation for the identification of *Anopheles* species in the Amazonas region of Peru. Although the sample size is limited, the detection of *Plasmodium* DNA in *An. benarrochi* B mosquitoes collected from native communities, combined with their anthropophilic and domestic behavior, as well as the rising malaria cases in these communities, provides strong evidence supporting *An. benarrochi* B’s potential role as a vector of Plasmodium in the region.

## Figures and Tables

**Table 1:** Identities of anopheline specimens collected in Rio Santiago district, Condorcanqui province, 2022.

Site	Date of Collection	UTM X	UTM Y	T° (°C)	RH (%)	No.	*Cox1* Id.
NE	March 2022	196,280.9	9,581,345.6	27	75	5	*An. benarrochi* B
CH	March 2022	199,060.7	9,587,806.4	27	87	1	*An. benarrochi* B
AP	March and May 2022	195,051.2	9,592,391.3	26	75	50	*An. benarrochi* B
	March 2022			26	75	2	*An. triannulatus*
	May 2022			25.3	87	2	*An. costai*
	May 2022			25.3	87	1	*An. nimbus*
CT	September 2022	198,182.6	9,566,709	25.3	87	5	*An. benarrochi* B

No., Number of specimens; *Cox1* Id. mtDNA *Cox1* sequence identification; NE, Nueva Esperanza; CH, Chapiza; AP, Alianza Progreso; CT, Caterpiza.

## Data Availability

The original contributions presented in this study are included in the article. Further inquiries can be directed to the corresponding author.
